# Deafness Cured by the Endermic Use of Morphia

**Published:** 1843-01

**Authors:** 


*Deafness cured by the endermic use of Morphia.*—Dr. Hoebeke relates in the *Archives de Med. Belge,* the following case of deafness, the cure of which he ascribes to the endermic use of morphia. A lady had become so deaf after an attack of fever that she could not distinguish a word, unless it was bawled into her ear by applying the mouth close to it. But along with the deafness there was always an incessant noise in the ears—at one time like the hissing of boiling water, at other times like the roaring of a hundred voices together—which was often so distressing as to cause headache and confusion of ideas:—these feelings were always worse when the head was on the pillow. There was a quantity of wax in the ears; but no relief was obtained when it was removed. Nothing irregular could be perceived either in the ears themselves or in the throat. Leeches were applied behind the ears, and emetics and purgatives given; but no relief followed. Supposing that the symptoms might be dependent upon some anomalous state of the nervous apparatus, a blister was applied behind each ear, and the excoriated surface was sprinkled with half a grain of sulphate of morphia. By the next day the noise and deafness on the left side had quite ceased, and on the right were much abated:—the headache, too, had disappeared.

As the unpleasant feelings still continued on the right side, a second blister was applied and treated in the same manner as before, with morphia:—the success was decided, and the patient was quite freed of all her annoyances.

[We opine the blistering had quite as much to do with the cure as the morphia.]—*American Journal of Med. Sci.*

